# Long-Term Clinical Outcomes After Ultrasound-Guided Cervical Retrolaminar Block in Patients with Cervical Radiculopathy

**DOI:** 10.3390/jcm15134965

**Published:** 2026-06-25

**Authors:** Uri Hochberg, Adi Lichtenstein, Wisam Zbede, Ahmad Taher, Jesus de Santiago, Silviu Brill, Morsi Khashan

**Affiliations:** 1 Institute of Pain Medicine, Division of Anesthesiology, Tel Aviv Sourasky Medical Center, Tel Aviv 64239, Israelsilviubril@gmail.com (S.B.); 2 Gray Faculty of Medical and Health Sciences, Tel Aviv University, Tel Aviv 69978, Israel; 3 Orthopedic Division, Tel Aviv Sourasky Medical Center, Tel Aviv 64239, Israel; 4 Pain Unit, Department of Anesthesia and Chronic, Hospital Quirónsalud de Tenerife, 38006 Santa Cruz de Tenerife, Spain; 5 Spine Surgery Unit, Neurosurgical Department, Orthopedic Division, Tel Aviv Sourasky Medical Center, Founder Building, 5th Floor, 6 Weizmann Street, Tel Aviv 64239, Israel

**Keywords:** radiculopathy, cervical spine, neck pain, retrolaminar injection, recurrence, decompression surgery

## Abstract

**Background/Objectives:** Cervical radiculopathy is a frequent cause of pain, often leading to disability, reduced quality of life, and significant healthcare utilization. Cervical epidural steroid injections are widely used, though safety concerns have been reported. Ultrasound-guided cervical retrolaminar block (RLCB) is a potential alternative. The purpose of this study was to evaluate the long-term clinical trajectory after ultrasound-guided cervical retrolaminar block, including pain outcomes, patient-reported improvement, and the rate of subsequent cervical spine surgery. **Methods:** This is a retrospective cohort analysis that was conducted at the Pain and Spine Surgery units in a single center. We included 121 patients with cervical radiculopathy treated between January 2020 and September 2022 (mean age 49.4 ± 11.1 years; 51.2% male). All patients underwent RLCB. *s*: Primary outcome measures were subsequent cervical decompressive surgery and composite pain response (≥2-point absolute and ≥50% relative NRS reduction). Secondary outcome measures included recurrence, analgesic use, global rating of change (GRC), satisfaction, willingness to repeat, and safety. Baseline data was extracted from records; structured follow-up interviews were conducted at two years. **Results:** At two years, 9.1% required surgery, and 57.9% achieved composite pain response; 74.4% reported ≥2-point NRS reduction. GRC scores showed improvement (mean 5.0 ± 3.4), with 37% reporting “very much better.” Satisfaction was high, with 70.2% willing to repeat. Pain recurred in 71.1% but persisted in 28.9%. No major complications occurred; minor events were reported in 6.6%. Outcomes were less favorable in patients with pre-injection pain duration ≥1 year. **Conclusions:** In this retrospective cohort, cervical RLCB was associated with sustained patient-reported improvement, high satisfaction, and a 9.1% observed subsequent surgery rate at two years. These findings are hypothesis-generating and require confirmation in prospective controlled studies.

## 1. Introduction

Cervical radiculopathy is a common cause of neck and arm pain, often leading to disability, reduced quality of life, and significant healthcare utilization. Cervical radiculopathy has an incidence of 1.79 per 1000 person-years [1,2].

Conservative treatments typically include modalities such as physical therapy, medication, and cervical epidural steroid injections (ESIs). ESIs are frequently used when initial measures fail; however, serious complications, including severe neurological injuries, have been reported, particularly when using the transforaminal route with particulate steroids [3,4].

Ultrasound-guided paraspinal inter-fascial plane blocks (UGPIPBs) recently emerged as a potential option for pain management in shoulder and cervical spine procedures [5,6,7]. Certain types of UGPIPB, such as the retro-laminar cervical block (RLCB), have shown preliminary effectiveness in managing cervical radicular pain [8,9]. RLCB is a technique that targets the space posterior to the cervical lamina. However, data regarding the long-term durability of pain relief and the potential impact on subsequent surgical intervention remain limited, accounting for the fact that long-term follow-up is crucial to assess symptom durability, detect delayed complications, and guide clinical decision making.

Despite growing interest in paraspinal interfascial plane blocks as alternatives to neuraxial injections, published data on RLCB remain limited to short-term and pilot studies. The clinical need for long-term data is particularly pressing given safety concerns associated with cervical transforaminal approaches and the risk of delayed complications. Establishing the durability of pain relief and the rate of surgical conversion at two years is a meaningful step toward understanding whether RLCB may have a role in the long-term management of cervical radiculopathy.

We conducted a two-year retrospective cohort study of patients treated with ultrasound-guided RLCB. The primary objectives were to determine the proportion achieving a composite pain response (≥2-point absolute and ≥50% relative NRS reduction) and the rate of subsequent cervical spine surgery. Secondary objectives included determination of pain recurrence, analgesic use, patient satisfaction, and safety. We hypothesized that cervical retrolaminar block may produce sustained pain relief in a substantial proportion of patients with cervical radiculopathy.

## 2. Methods

### 2.1. Study Design and Setting

This retrospective cohort study was conducted at the Pain Institute Center and the Spine Surgery Unit of a tertiary university-affiliated medical center. The study was approved by the Tel Aviv Sourasky Medical Center Institutional Review Board (Helsinki committee), approval number 0515-19-TLV, granted on 29 October 2023. All patients had provided written informed consent before the procedure and verbal consent for study participation.

### 2.2. Patients

Patients who underwent ultrasound-guided cervical retrolaminar block (RLCB) between January 2020 and September 2022 were included. This retrospective cohort included all consecutive eligible patients treated during the defined period. No a priori sample size calculation was performed; the study is best interpreted as an estimation study rather than a powered confirmatory trial. Referral was made by treating spine surgeons.

Each patient received a diagnosis of cervical radiculopathy from a single spine surgeon during evaluation at the spine surgery clinic before injection referral. Across the full cohort, five spine surgeons contributed to the diagnostic assessment. The diagnosis was based on a combination of clinical history, physical examination, and imaging findings. Clinical presentation included symptoms consistent with cervical radiculopathy. These were defined as neck and/or upper-extremity pain, sensory disturbance, attributable to compression or irritation of a cervical nerve root. Physical examination findings included a dermatomal distribution of symptoms with or without positive provocative maneuvers, such as the Spurling test. Magnetic resonance imaging (MRI) was used to confirm a structural lesion corresponding to the symptomatic nerve root, including disk herniation, foraminal stenosis, uncovertebral or facet joint hypertrophy, or a combination of these pathologies. The diagnosis was confirmed when clinical and radiographic findings were concordant.

Inclusion criteria: Charts were selected for inclusion if the following criteria were documented at the time of the procedure: age ≥ 18 years, cervical radicular pain with NRS ≥ 4, absence of significant motor weakness or clinical myelopathy requiring surgical decompression, and failure of prior conservative treatments. Exclusion criteria: Exclusion criteria were selected to reflect standard contraindications to corticosteroid injection procedures: allergy to steroids or amide anesthetics represents a direct safety contraindication; pregnancy and breastfeeding were excluded due to the corticosteroid component and the absence of safety data in these populations. These criteria align with those reported in comparable cervical injection studies.

### 2.3. Data Collection

Patients were identified from a prospectively maintained procedural registry at the Pain Institute. All patients who underwent RLCB during the study period were screened for eligibility by chart review. Eligible patients were contacted by telephone for structured interview; a minimum of three contact attempts were made. Participation was voluntary, and patients provided verbal informed consent prior to interview.

Baseline demographic, clinical, and imaging data (age, sex, prior cervical surgery, pain duration, cervical pathology, injection details) were systematically collected from medical records.

Structured telephone interviews were conducted by trained research staff. A minimum of three contact attempts were made before classifying a patient as lost to follow-up. Patients who declined participation were recorded separately. For items with missing responses, the denominator reported reflects available data only; missing values were not imputed. The phone interviews gathered post-procedural data:

1. Recurrence of Cervical Radicular Pain: Patients were categorized into three groups based on their responses regarding pain recurrence:
  - No recurrence.
  - Sporadic—one or more painful episodes occurring after the resolution of the initial episode treated with injection.
  - Persistent—ongoing, constant painful episodes that remain present at the time of follow-up.
2. Management of Recurrence: In case of recurrence, how was it managed?
3. Willingness to Repeat Procedure: On a scale of 1 to 5 (1 = “no way” and 5 = “absolutely yes”), would you consider receiving RLCB again if needed?
4. Satisfaction scale: On a scale of 1 to 5 (1 = “very dissatisfied” and 5 = “very satisfied”), what was your overall impression of the procedure?
5. Adverse effects or complications.
6. Current pain medications.
7. Global Rating of Change (GRC) questionnaire (−7 = “very much worse” to +7 = “very much better”).
8. Subsequent cervical spine surgery.

### 2.4. Procedure

All procedures were performed in the prone position under ultrasound and Doppler guidance. A 22G needle was advanced in-plane to the posterior lamina at the target level, identified by counting upward from the C7 transverse process. Fluoroscopy was used for confirmation only. After injection of 1 mL iodexol (270 mg/mL) to exclude intravascular or intrathecal spread, 4 mL of a solution containing 1% lidocaine and 10 mg of dexamethasone was administered. All procedures were performed by proficient pain physicians.

### 2.5. Outcome Measures

The primary outcomes were 1. Incidence of subsequent cervical decompressive surgery (after the index block) and 2. Composite pain response, defined as both an absolute reduction in NRS ≥ 2 points [10] and a relative reduction of ≥50%.

Secondary outcomes included individual pain response (absolute or relative response), total number of injections, pain recurrence and analgesic reliance at follow-up, GRC score, and patient-reported satisfaction as measured by the satisfaction scale and willingness to repeat the injection. Safety outcomes included any adverse events or complications reported during follow-up.

### 2.6. Statistical Analysis

Continuous variables are presented as means (±SD), and categorical variables as counts and percentages. Group differences were assessed with logistic regression, and results are reported as odds ratios with 95% confidence intervals. Exploratory subgroup analyses were performed according to pre-injection pain chronicity (≥1 year vs. <1 year). Chronicity variables (pre-injection chronicity and current persistent pain phenotype) were also evaluated as potential predictors in regression models. Outcome rates were compared across chronicity strata using logistic regression, and proportions are reported with Wilson 95% confidence intervals. Penalized logistic regression with an L1 penalty (LASSO) was applied to reduce overfitting. The regularization parameter (λ) was selected by 10-fold cross-validation minimizing binomial deviance. Candidate predictors included age, sex, baseline NRS, pain duration/chronicity category, prior cervical surgery, treated cervical level, laterality, and imaging pathology. Variables with non-zero coefficients at the selected λ were retained. Model performance was evaluated by cross-validated AUC. Given the small number of surgical events (*n* = 11), surgery models were exploratory only and were not used for confirmatory inference. Model performance was evaluated with cross-validated area under the receiver operating characteristic curve (ROC–AUC) and accuracy. All analyses were two-sided, with *p* < 0.05 considered statistically significant. Statistical analyses were conducted by an external, independent statistician.

## 3. Results

### 3.1. Study Population

Of 151 patients treated with cervical retrolaminar injections between January 2020 and September 2022, 30 were not evaluable (18 lost to follow-up, 8 declined participation, and 4 ineligible). No meaningful differences in age, sex, or baseline NRS were identified between included and excluded patients, though this comparison is underpowered given the small number of non-evaluable cases. A total of 121 patients were included in the analysis. The mean age was 49.4 ± 11.1 years and 62 (51.2%) were male patients. Other baseline variables are shown in Table 1.

### 3.2. Primary Outcomes

Cervical surgery after injection was required in 11 (9.1%) patients and a composite pain response was achieved in 70 (57.9%) patients (Table 2).

### 3.3. Secondary Outcomes

Absolute NRS reduction of more than two points was found in 90 (74.4%) patients. The GRC demonstrated a broad distribution, with a mean score of 5.0 ± 3.4 (Table 2). The majority reported improvement, with 44 (37.0%) rating “+7 = a very great deal better” and many others scoring in the high positive range. Recurrent pain was reported by most patients, but only 35 (28.9%) reported persistent recurrent pain. Among those with recurrence, 18 (15.0%) underwent a repeat injection and 11 (9.1%) required surgery. Most patients reported no current medication use at follow-up. Medication use was more frequent among patients who subsequently underwent surgery after injection (55.6% vs. 23.4%; *p* = 0.049).

**Figure 1:**
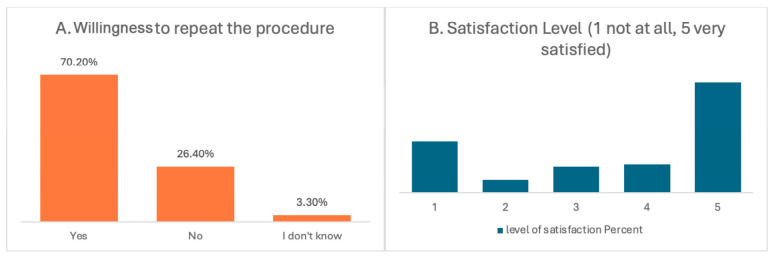
Patient-reported satisfaction and acceptance.

Looking at patients’ reported satisfaction, 85 of 121 patients (70.2%) indicated they would repeat the injection, whereas 32 (26.4%) declined and 4 (3.3%) were uncertain (Figure 1, Panel A). Satisfaction ratings were skewed toward higher scores, with 58 patients (47.9%) reporting “very satisfied” (score 5) and a mean satisfaction score of 3.58 (SD, 1.64) (Figure 1, Panel B).

Patient satisfaction was assessed using a 5-point Likert scale, ranging from 1 (*not at all satisfied*) to 5 (*very satisfied*). Acceptance was evaluated by the reported willingness to undergo the procedure again, scored from 1 (*no way*) to 5 (*absolutely yes*). Bars show the distribution of responses across the study cohort. Percentages are based on the number of patients with available data.

### 3.4. Safety

No major or delayed complications were observed during follow-up. Overall, eight patients (6.6%) reported minor adverse effects: four reported transient injection-site pain, two patients reported palpitations and listlessness in the days after injection, one patient experienced nausea and vomiting immediately post-procedure (vasovagal reaction), and one patient reported transient facial numbness. All events resolved spontaneously without intervention. The safety profile was consistent across patient subgroups, irrespective of subsequent surgical intervention or development of chronic pain.

### 3.5. Chronicity

**Figure 2:**
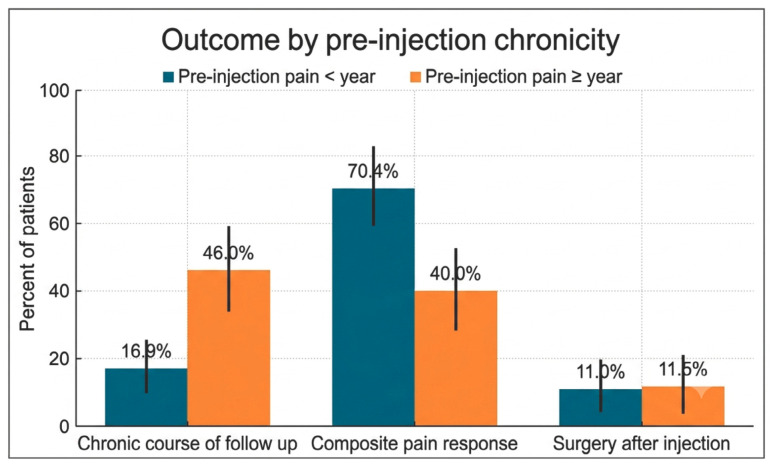
Outcomes stratified by chronicity.

In exploratory analyses, patients with pre-injection pain duration ≥1 year were more likely to have a persistent pain course than those with shorter duration (46.9% vs. 16.9%; OR, 4.35; *p* = 0.0006). Composite response rates were also lower (40.0% vs. 70.4%; *p* < 0.001) (Figure 2). These findings suggest that chronicity may influence outcomes, but the study was not powered to establish it as a definitive predictor.

Panel A shows outcomes according to pre-injection chronicity (pain duration ≥1 year vs. <1 year). Panel B shows outcomes according to the current chronic phenotype (chronic pain at follow-up vs. non-chronic). Bars indicate the proportion of patients with a chronic pain course, composite pain response, or subsequent surgery. Error bars represent Wilson 95% confidence intervals.

### 3.6. Predictors of Pain Response

Beyond chronicity, we did not identify consistent baseline predictors of a composite pain response or subsequent cervical surgery. Multivariable and penalized regression did not reveal stable independent predictors, with wide confidence intervals reflecting the modest sample size and few surgical events. These results suggest that although chronicity may be associated with outcome patterns, no demographic or clinical features reliably predicted response in this cohort. Larger studies will be needed to clarify the prognostic role of chronicity and other variables.

## 4. Discussion

In this retrospective cohort study with a minimum follow-up of two years, ultrasound-guided cervical retrolaminar block was associated with meaningful long-term pain reduction, high patient satisfaction, and a relatively low rate of subsequent cervical spine surgery. Approximately 58% of patients achieved a composite pain response, nearly three-quarters reported clinically meaningful pain reduction, and only 9% ultimately required surgical decompression during the observed follow-up period. These findings suggest that RLCB may represent a durable non-surgical treatment option for selected patients with cervical radiculopathy who do not respond to conservative therapy.

Long-term follow-up after a therapeutic procedure is essential as early benefits of interventional pain management may fade and longer-term complications may emerge [11,12]. This is particularly relevant due to the need to monitor rare but serious incidents such as infections [13,14,15]. Hence, our study focused on efficacy, safety, and patient experience—fundamental factors for assessing new techniques.

### 4.1. Efficacy

Since single pain scales incompletely capture the multidimensional impact of pain, we evaluated outcomes using several complementary domains including pain intensity, global improvement, medication use, and subsequent surgical intervention [16]. The GRC is widely used to assess patient-perceived changes in health status, offering valuable insights into treatment outcomes and patient-centered care. However, its interpretation can be influenced by baseline health and individual cognitive biases [17,18]. Therefore, we combined NRS and GRC with objective indicators, including surgical rates and medication changes, to ensure a more complete assessment of treatment effectiveness and durability.

Despite the overall positive analgesic effect reported, 9.1% of patients required cervical surgery, mostly those patients suffering from longer pain duration and spinal cord compression. This rate is slightly lower than reported after cervical epidural steroid injections, where surgical conversion ranged from 11% at six-month follow-up to 14–22% within one to five years, with some cohorts reporting 19–37% [19,20]. Our long-term results therefore support RLCB as a potential ‘surgery-sparing’ option and provide evidence showing that RLCB may provide sustained value with a slightly lower surgical rate compared with outcomes reported for cervical epidural steroid injections (CESIs). This points to RLCB as a safe alternative for patients who have failed conservative therapy.

We found that most patients (71.1%) experienced prolonged pain relief and did not progress to persistent pain following RLCB. Achieving this outcome typically required fewer than two injections. These results suggest that RLCB may provide meaningful benefits. Notably, among patients with recurrence, nearly half required no additional treatment, reflecting the procedure’s efficacy in reducing dependence on subsequent interventions.

The GRS scores in our cohort align with prior research comparing cervical interlaminar epidural steroid injections and cervical transforaminal epidural steroid injections. In this study, around 53–54% of patients in both groups reported being “much improved” or “very much improved” at six months [21], demonstrating comparable patient-reported benefits to an established technique. This similarity supports the potential of RLCB as an equally effective alternative for managing cervical radiculopathy, while potentially avoiding epidural risks.

Our findings highlight two prominent trends in current use of medication. Firstly, opioid exposure was mostly absent in this cohort (used by only one (0.8%) patient), highlighting the cautious approach of physicians in prescribing these agents for chronic, non-acute pain. Secondly, a substantial sub-section of the cohort reported using medical cannabis (11.6% of patients), which is consistent with its emerging but still debated role in multimodal pain management [22]. Interestingly, at the two-year follow-up, almost three-quarters of patients (73.6%) reported no regular analgesic use, while the remainder continued primarily with non-opioid regimens (14.0%) such as OTC analgesics, SNRIs, Gabapentinoids, and tricyclic antidepressants (e.g., amitriptyline). However, as baseline medication use was not systematically quantified prior to injection, this should be interpreted as a cross-sectional observation rather than evidence of pharmacological reduction attributable to the procedure.

As for patient satisfaction, patients reported high levels of satisfaction, with 60.3% experiencing positive outcomes and 70.2% indicating willingness to undergo the procedure again if necessary. This broad acceptance of RLCB may be due to the procedure efficacy and, in part, to its low rate of adverse events and practical advantages, such as the absence of a sedation requirement, brief procedural duration, and quick discharge post-procedure.

### 4.2. Safety

The RLCB approach demonstrated a favorable safety profile: no major or delayed complications were observed, and only 6.6% of patients reported minor, self-limited adverse events. The favorable safety profile likely reflects the anatomical characteristics of the technique, which targets the posterior lamina while avoiding the epidural space and major vascular structures. The technique is technically simpler, avoids dura and neuraxial structures, thereby reducing risks. Our findings are consistent with prospective evaluations of ultrasound-guided paraspinal techniques such as the continuous multifidus muscle block, which have likewise reported only transient, minor adverse effects and no serious complications [23]. Taken together, these observations support the favorable safety profile of RLCB, particularly for patients at elevated risk with more invasive approaches.

At the time of the study, our institutional protocol included fluoroscopic confirmation and contrast injection prior to drug administration, reflecting our dual-confirmation standard of care during the initial adoption phase of RLCB. This practice has since been discontinued as operator experience matured.

### 4.3. Predictors of Pain Response and Chronicity

In this study, we did not identify any reliable baseline predictors of a clinically meaningful pain response or of subsequent cervical surgery. Prior studies have reported mixed results. Some studies found that the likelihood of treatment success is reduced by longer pain duration, spinal stenosis, or neuropathic pain features [24], while others identified etiology (disk herniation vs. bony foraminal stenosis) as the only significant factor [25]. However, others reported no consistent associations between baseline characteristics and treatment response [26]. In our cohort, chronicity showed an association with outcome patterns in preliminary analyses, but it did not transpire as a stable independent predictor. These conflicting findings suggest that commonly assessed clinical variables may have limited prognostic value, that outcomes are probably influenced by multifactorial, patient-specific factors, and that larger, prospective cohorts will be required to detect truly valuable predictors.

### 4.4. Broader Management Considerations

Cervical radiculopathy is a multidimensional pain condition, and RLCB may represent one component of a broader pain-management strategy. In our cohort, 28.9% of patients had persistent recurrent pain at two-year follow-up, highlighting the need to address chronification risk alongside nociceptive and neuropathic pain generators. Recovery may be supported by patient education, graded return to activity, ergonomic adaptation, cervical strengthening, and supervised physiotherapy when appropriate. Evidence suggests that psychological factors shape pain experience; positive affect has shown analgesic effects in experimental pain models and, by broadening attention and promoting adaptive coping, may reduce catastrophizing and support improved self-management in chronic pain populations, while resilience may be a protective factor in adapting to chronic pain [27,28]. Positive psychology interventions have been associated with reductions in pain intensity, depressive symptoms, and catastrophizing in chronic pain patients [29]. Combining interventional treatment with rehabilitation and psychological support warrants prospective evaluation in this patient group.

### 4.5. Generalizability Beyond Local Context

While the study involved only one single center, the clinical implications might be applicable to larger populations of cervical radiculopathy patients. The good safety profile associated with RLCB compared to epidural ESI, along with patient satisfaction and lack of requirement for subsequent surgery among patients enrolled in the study, indicates that cervical retrolaminar block might be considered as an option for selected patients in different healthcare settings. Moreover, the fact that the procedure is possible to perform under ultrasound guidance without the need for sedation and minimal resource use makes it even more appealing in today’s times, when many healthcare systems have to cope with ever-growing financial constraints and lack of specialists. However, factors such as referral pattern, patient expectations, financing of healthcare and surgery threshold might differ from country to country, which means that generalizability of our results needs further evaluation in a prospective study.

### 4.6. Limitations

This study has several limitations. Its retrospective, single-center design may have introduced selection and reporting biases, and patient-reported outcomes are subject to recall error. Sample size is modest, which reduces the ability to carry out subgroup analyses and restricts generalizability. An important difficulty is separating the effects of the corticosteroid component on the reduction in pain from the effects of the procedure itself. All the subjects in this study received dexamethasone, and any pain reduction could be largely attributed to its anti-inflammatory properties, regardless of which anatomic area was treated. Comparator studies with the same steroids are necessary to separate the mechanical effects [30,31]. Standardized functional disability measures such as the Neck Disability Index (NDI), which was not used in this study, could have provided further insight into the procedure’s impact. Patient-reported outcomes were collected via structured telephone interview at two years. Because post-procedure pain intensity was obtained retrospectively rather than prospectively at fixed intervals, NRS change scores should be interpreted cautiously. Current pain status may disproportionately anchor retrospective assessments, and recall bias cannot be excluded. Finally, approximately 20% of the initial cohort (30/151) was not evaluable at the two-year mark (18 lost to follow-up, 8 declined, 4 ineligible). Patients lost to follow-up may disproportionately include those with poor outcomes or who sought care elsewhere, potentially inflating estimates of treatment success and underestimating surgical conversion rates.

A key strength of this study is the long-term (2-year) follow-up, which offers insight into the durability of treatment response and safety in a real-world cohort. These findings support the need for prospective, comparative studies which can directly evaluate and compare RLCB against cervical epidural steroid injections to establish relative effectiveness and guide clinical practice.

## 5. Conclusions

Ultrasound-guided cervical RLCB was associated with sustained patient-reported improvement, high levels of satisfaction, and a low observed rate of subsequent cervical surgery at two-year follow-up. Outcomes appeared less favorable among patients with longer symptom duration. Given the retrospective observational design and the absence of a control group, these findings should be considered hypothesis-generating and require confirmation in prospective controlled studies before definitive conclusions regarding effectiveness or surgery-sparing effects can be drawn.

## Figures and Tables

**Table 1:** Patient characteristics.

Total	121
**Age**	49.4 ± 11.1
**Male**	62 (51.2%)
**Pain duration before injection**	
<3 months	30 (25.6%)
3–6 months	17 (14.5%)
6–12 months	20 (17.1%)
1–3 years	37 (31.6%)
>3 years	13 (11.1%)
**Cervical surgery before the injection**	10 (8.3%)
**NRS score pre-injection**	7.4 ± 1.4
**NRS score post-injection**	3.4 ± 2.9
**Cervical level**	
C4	2 (1.7%)
C5	25 (20.8%)
C6	84 (70.0%)
C7	9 (7.5%)
**Cervical sides**	
Right	53 (44.2%)
Left	52 (43.3%)
Bilateral	15 (12.5%)
**Pathology**	
Nerve root compression	89 (80.2%)
Myelomalacia without myelopathy	5 (4.5%)
Cord compression without myelopathy	11 (9.9%)
Bulging disk without nerve compression	3 (2.7%)

Plus–minus values are means ± SD. Percentages are based on the number of patients with available data. NRS = Numerical Rating Scale (0–10, with higher scores indicating greater pain).

**Table 2:** Outcomes.

Total	121	95% Confidence Interval
**Primary Outcomes**		
Cervical Surgery (after injection)	11 (9.1%)	4.2–15.1
Composite Pain Response	70 (57.9%)	49.6–68.1
**Secondary Outcomes**		
NRS Reduction ≥ 2 Points (absolute)	90 (74.4%)	68.1–83.2
NRS Reduction ≥ 50% (relative)	70 (57.8%)	50.4–68.9
Recurrence Pattern		
No Recurrence	50 (41.3%)	32.0–49.6
Sporadic	36 (29.8%)	22.7–38.7
Persistent	35 (28.9%)	21.0–37.0
Management of Recurrence		
None	55 (45.8%	37.0–54.6
Conservative	36 (30.0%)	21.8–38.7
Injection	18 (15.0%)	9.2–21.8
Surgery	11 (9.1%)	3.4–14.3
Current Medications		
None	89 (73.6%)	67.2–81.5
Non-opioids	17 (14.0%)	8.4–21.0
Cannabis	14 (11.6%)	5.9–16.8
Opioids	1(0.8%)	0–2.5
Total Number of Injections	1.7 ± 0.8	
Global Rating of Change (GRC)	5.0 ± 3.4	

NRS = Numerical Rating Scale (0–10, with higher scores indicating greater pain). GRC = Global Rating of Change (range −7 to +7, with higher scores indicating greater improvement). Composite Pain Response is defined as both an absolute reduction of ≥2 points and a relative reduction of ≥50% on the NRS. Non-opioids include over-the-counter analgesics and adjuvant medications (e.g., serotonin–norepinephrine reuptake inhibitors, gabapentinoids, and tricyclic antidepressants). Total number of injections refers to the mean number of retrolaminar cervical block procedures per patient. Management of recurrence data available for 120/121 patients; one patient has missing data for this item.

## Data Availability

The data presented in this study are available on request from the corresponding author.
